# The OPR gene family: bioinformatics characterization and functional implications for soybean seed deterioration

**DOI:** 10.3389/fpls.2026.1880971

**Published:** 2026-08-11

**Authors:** Zhonglu Yang, Zhihui Shan, Dong Cao, Bei Han, Shuilian Chen, Songli Yuan, Haifeng Chen, Xia Li, Xin’an Zhou

**Affiliations:** Oil Crops Research Institute, Chinese Academy of Agricultural Sciences, Wuhan, China

**Keywords:** *OPR* gene family, soybean deterioration, bioinformatics analysis, jasmonic acid, jasmonoyl-isoleucine, synteny analysis

## Abstract

**Introduction:**

12-oxophytodienoate reductases (OPRs) are vital enzymes for jasmonic acid (JA) biosynthesis, which mediate plant growth, development and stress defense. Characterizing the soybean OPR gene family is essential for breeding soybean cultivars with strong seed storage stability.

**Methods:**

Genome-wide identification and systematic analysis of soybean OPR genes were performed in this study. Twelve OPR members were identified and renamed according to their chromosomal positions, and their chromosomal distribution patterns were analyzed. A multi-species phylogenetic tree of OPR proteins was constructed, along with analyses of conserved motifs and gene structures. Cis-acting elements were predicted to explore hormone-responsive regulatory sequences, and the expression correlation between GmOPR11 and seed deterioration resistance was analyzed under different sowing dates.

**Results:**

A total of 12 GmOPR genes were identified and unevenly scattered on soybean chromosomes. Phylogenetic tree, conserved motif and gene structure analyses clarified the evolutionary features of the OPR family. Cis-element prediction showed that abscisic acid (ABA)- and methyl jasmonate (MeJA)-responsive cis-elements occupied the largest proportion. The expression of GmOPR11 was significantly negatively correlated with soybean seed deterioration resistance across different sowing treatments.

**Discussion:**

This negative correlation may be caused by excessive accumulation of jasmonic acid (JA). Excess JA could disrupt endogenous hormone homeostasis and trigger reactive oxygen species production, which in turn accelerates soybean seed deterioration. This study systematically characterizes the evolutionary patterns and biological functions of the soybean OPR family, laying a theoretical foundation for breeding soybean varieties with enhanced seed storage stability.

## Introduction

Soybean (*Glycine max*), an annual herbaceous species belonging to the Fabaceae family, occupies a pivotal position in the global agricultural economy. Native to China, it has been widely cultivated across the world, with major production regions distributed in the United States, China, Brazil, Argentina, and India (Guo et al., 2022). In recent years, the global soybean cultivation area has reached approximately 130 million hectares, with a total yield of around 360 million tons. The agricultural and economic significance of soybean stems from its high nutritional value, as it contains 40%–45% protein and 20%–22% oil, and serves as a primary source of plant-based protein and vegetable oil globally (Rao et al., 2023). Furthermore, soybean is rich in dietary fiber, vitamins, and minerals, and thus finds extensive applications in food processing, animal feed production, and various industrial sectors (Al Loman and Ju, 2017; Elhalis et al., 2024; Rahman et al., 2021).

However, soybean seeds are highly prone to deterioration during post-harvest storage, especially under adverse environmental conditions (Rao et al., 2023). Seed deterioration is a complex physiological and biochemical process characterized by the gradual decline in seed quality, which is manifested as reduced germination capacity, impaired seedling vigor, and diminished nutritional integrity (Pinheiro et al., 2021; Kafer et al., 2025). Tropical and subtropical regions with high humidity and temperature are particularly susceptible to severe soybean seed deterioration (Baributsa and Baoua, 2022). Key factors contributing to soybean seed deterioration include oxidative stress-induced lipid peroxidation (Lin et al., 2022), protein denaturation (Min et al., 2017; Yin et al., 2016), DNA damage (Fleming et al., 2019), as well as microbial infection caused by structural damage to the seed coat surface, which collectively accelerate seed decay. Thus, elucidating the molecular regulatory mechanisms governing seed deterioration and identifying key genes involved in anti-deterioration pathways are critical for improving soybean seed storage performance.

In our preliminary studies, integrated transcriptomic and metabolomic analyses identified 12-oxophytodienoate reductase (*OPR*) as one of the candidate genes with the highest correlation to soybean seed deterioration. *OPR* is a key enzyme in the jasmonic acid (JA) biosynthesis pathway, catalyzing the reduction of 12-oxophytodienoic acid (OPDA) to 3-oxo-2-(2’-pentenyl)-cyclopentane-1-octanoic acid (OPC-8:0), a rate-limiting step in JA biosynthesis (Schaller et al., 2000; Wang et al., 2022). As an essential phytohormone, JA regulates multiple physiological processes in plants, including growth, development, and responses to biotic and abiotic stresses (Ruan et al., 2019). Overexpression of OPR family genes generally strengthens plant stress resistance, yet their underlying regulatory mechanisms differ substantially across gene members and plant species. For instance, wheat *TaOPR1* confers salt tolerance via activating ABA signal transduction and enhancing ROS scavenging capacity, and this salt-resistance operates in a JA-independent manner (Dong et al., 2013). In contrast, cotton *GhOPR9* elevates plant resistance against *Verticillium* wilt through canonical JA-dependent signaling cascades (Liu et al., 2020). Meanwhile, heterologous expression of *Arabidopsis AtOPR3* in wheat triggers a JA biosynthesis positive feedback loop to accumulate endogenous JA; the elevated JA pool simultaneously improves freezing tolerance, delays floral transition, and suppresses leaf senescence (Pigolev et al., 2018). Collectively, the broad functional divergence within the *OPR* gene family offers diverse genetic resources and regulatory strategies for stress-tolerant crop molecular breeding. This suggests their potential role in maintaining seed quality. However, the specific functions and underlying molecular mechanisms of *OPR* in soybean seed deterioration remain poorly understood and require systematic investigation.

The present study aimed to comprehensively analyze the 12-oxophytodienoate reductase gene family in soybean. Using bioinformatics tools, we identified and characterized *OPR* gene family members, analyzed their gene structures, phylogenetic relationships, and tissue-specific expression patterns. Additionally, functional validation was conducted using physiological and biochemical assays to clarify the roles of *OPR* genes in mediating soybean seed deterioration.

## Materials and methods

### Identification of *GmOPR* family genes

The whole genome sequence files and gene annotation files of *Arabidopsis*, soybean, and rice are from the Ensembl Plant Database (https://plants.ensembl.org/). The soybean coding sequence and target gene sequence were extracted using TBtools software. Gene screening local blast (E-value ≤ 1e-5) was completed using TBtools, the HMM of *OPR* domains was obtained from PFAM database (http://pfam.xfam.org/). Finally, predicted proteins were considered as *GmOPR* only if they contained NADH oxidase family (PF00724) conserved domains verified by TBtools.

### Sequence structure and phylogenetic analysis

The physical and chemical properties of the *GmOPRs*, including their molecular weights (k Da) and isoelectric points (pIs), were analyzed using ExPASy (https://web.expasy.org/protparam/). Subcellular localization of the *GmOPR* proteins was predicted using WoLF PSORT II. Motif pattern and gene structures were analyzed using TBtools. Conserved motifs of *GmOPRs* were predicted by MEME on line program (http://meme-suite.org/tools/meme). ClustalX2 software and the neighbor-joining method were used to construct a phylogenetic tree of the *OPR* genes in *Arabidopsis*, soybean, and rice. The tree was visualized using iTOL v6 (https://itol.embl.de/). All gene information used for multiple sequence alignment and phylogenetic tree construction, including transcript IDs, NCBI accession numbers, species names and protein sequences, is provided in Folder 1

### Gene location and expression patterns

The locations of the genes on the chromosomes were drawn using MG2C (http://mg2C.iask.in/mg2C_v2.1/). Collinearity analysis was performed using TBtools. The 2,000-bp upstream regions of the *OPR* genes were extracted using TBtools and submitted to PlantCARE (http://bioinformatics.psb.ugent.be/webtools/plantcare/html) to predict cis-acting elements in their promoter regions. Tissue expression heatmap data were obtained from the JGI Phytozome database (Phytozome (doe.gov)) and visualized using TBtools. The spatiotemporal expression profiles and corresponding figures were retrieved from the public soybean multi-omics database SoyOmics (https://ngdc.cncb.ac.cn/soyomics/transcriptome/tissues).

### Soybean growth and quantitative analysis of genes

The deterioration-resistant soybean cultivar Y17166 and the deterioration-susceptible cultivar Tianlong No.1, which were kept at Oil Crops Research Institute, Chinese Academy of Agricultural Sciences (OCRI-CAAS), were planted in the Wuhan region. Seven sowing date (SD) treatments were set in total: SD 1: April 8; SD 2: April 11; SD 3: April 26; SD 4: May 5; SD 5: May 15; SD 6: May 25; SD 7: June 5. The corresponding growth periods of the first four sowing dates were 114 d, 112 d, 100 d and 106 d, respectively, while the growth period of sowing date 5 was 109 d. At the beginning pod stage (R3), fully expanded trifoliate leaves from the upper part of the plant and young pods at the same node were collected from each SD treatment for qPCR analysis. All samples were immediately frozen in liquid nitrogen. For sowing dates 5 to 7, plants failed to set pods normally; therefore, only leaf samples were collected for these three treatments. Soybean plants from sowing dates 5 to 7 failed to form pods normally. This phenomenon is mainly caused by unsuitable photothermal conditions under late sowing. As a typical short-day crop, soybean exhibits a significantly shortened vegetative growth period under the long-day and high-temperature environment in early summer, resulting in insufficient biomass accumulation. Meanwhile, continuous high temperature during the flowering period disrupts floral organ development and pollination/fertilization, triggering severe flower and pod abscission and ultimately preventing normal pod formation. All figures were generated using GraphPad Prism software.

### Statistical analysis

All qRT-PCR expression data were obtained from at least three independent biological replicates with three technical replicates each, and the results are presented as mean ± standard error (SE). Statistical analyses were carried out using SPSS 26.0 software. One-way analysis of variance (ANOVA) followed by Duncan’s multiple range test was performed to evaluate the effects of genotype and sowing date on GmOPR11 expression, and a threshold of P<0.05 was considered statistically significant. All quantitative expression data plots were generated using GraphPad Prism software. **Supplementary Table 1**-**2** list the transcript IDs, gene names, coding sequence (CDS) sequences and corresponding primer sequences of all genes used for quantitative real-time PCR (qRT-PCR), including reference genes.

## Results

### Twelve *GmOPRs* display divergent physicochemical features

To identify the members of the *OPR* family in soybean, a comprehensive analysis of the soybean genome annotation was performed. Twelve *GmOPRs* were identified from the soybean genome and named *GmOPR1*-*GmOPR12* according to their chromosomal locations. Detailed information on *GmOPRs*, including gene name, gene ID, chromosomal localization, molecular weight (MW), theoretical isoelectric point (pI), aliphatic index, average hydrophilicity value, and subcellular localization, is listed in the table (**Table 1**). The 12 GmOPR proteins exhibited variations in physicochemical properties: their molecular weights ranged from 39.09 kDa (GmOPR11) to 45.19 kDa (GmOPR1). The pI values of GmOPRs spanned from 5.21 (GmOPR11) to 8.22 (GmOPR4 and GmOPR10), with nine members being acidic (pI < 7) and three basic (pI > 7), indicating that the majority are acidic proteins. In terms of other physicochemical properties, the aliphatic index of *GmOPRs* ranged from 72.32 (GmOPR7) to 86.56 (GmOPR9), reflecting differences in thermal stability among the proteins. All GmOPR proteins showed negative average hydrophilicity values (from –0.516 to –0.182), suggesting that they are generally hydrophilic. Subcellular localization analysis revealed that GmOPRs are distributed in multiple compartments: seven members are localized in the mitochondria, four in the cytoplasm, and one (GmOPR5) in the chloroplast, implying functional diversification across subcellular structures.

**Table 1:** Basic information of *OPR* family members in soybean.

Gene	Gene ID	Chr	MW (k Da)	pI	Aliphatic Index	Average hydrophilicity value	Subcellular localisation
*GmOPR1*	Glyma.01G235600	1	45.19	6.36	76.67	-0.432	Mitochondrial
*GmOPR2*	Glyma.06G016900	6	41.87	6.03	77.49	-0.342	Cytoplasmic
*GmOPR3*	Glyma.11G007600	11	41.52	6.12	84.12	-0.353	Cytoplasmic
*GmOPR4*	Glyma.13G109700	13	44.09	8.22	84.12	-0.286	Mitochondrial
*GmOPR5*	Glyma.13G109800	13	42.94	5.96	81.49	-0.231	Chloroplast
*GmOPR6*	Glyma.13G186200	13	41.01	5.96	81.34	-0.365	Cytoplasmic
*GmOPR7*	Glyma.14G223600	14	43.79	6.47	72.32	-0.516	Mitochondrial
*GmOPR8*	Glyma.15G223900	15	40.33	6.5	80.00	-0.409	Cytoplasmic
*GmOPR9*	Glyma.17G049900	17	42.05	6.34	86.56	-0.182	Mitochondrial
*GmOPR10*	Glyma.17G050000	17	44.19	8.22	84.6	-0.298	Mitochondrial
*GmOPR11*	Glyma.17G209900	17	39.09	5.21	83.44	-0.284	Cytoplasmic
*GmOPR12*	Glyma.19G057500	19	40.53	7.70	82.55	-0.323	Mitochondrial

### *GmOPRs* show lineage-specific expansion and interspecies conservation

**Figure 1:**
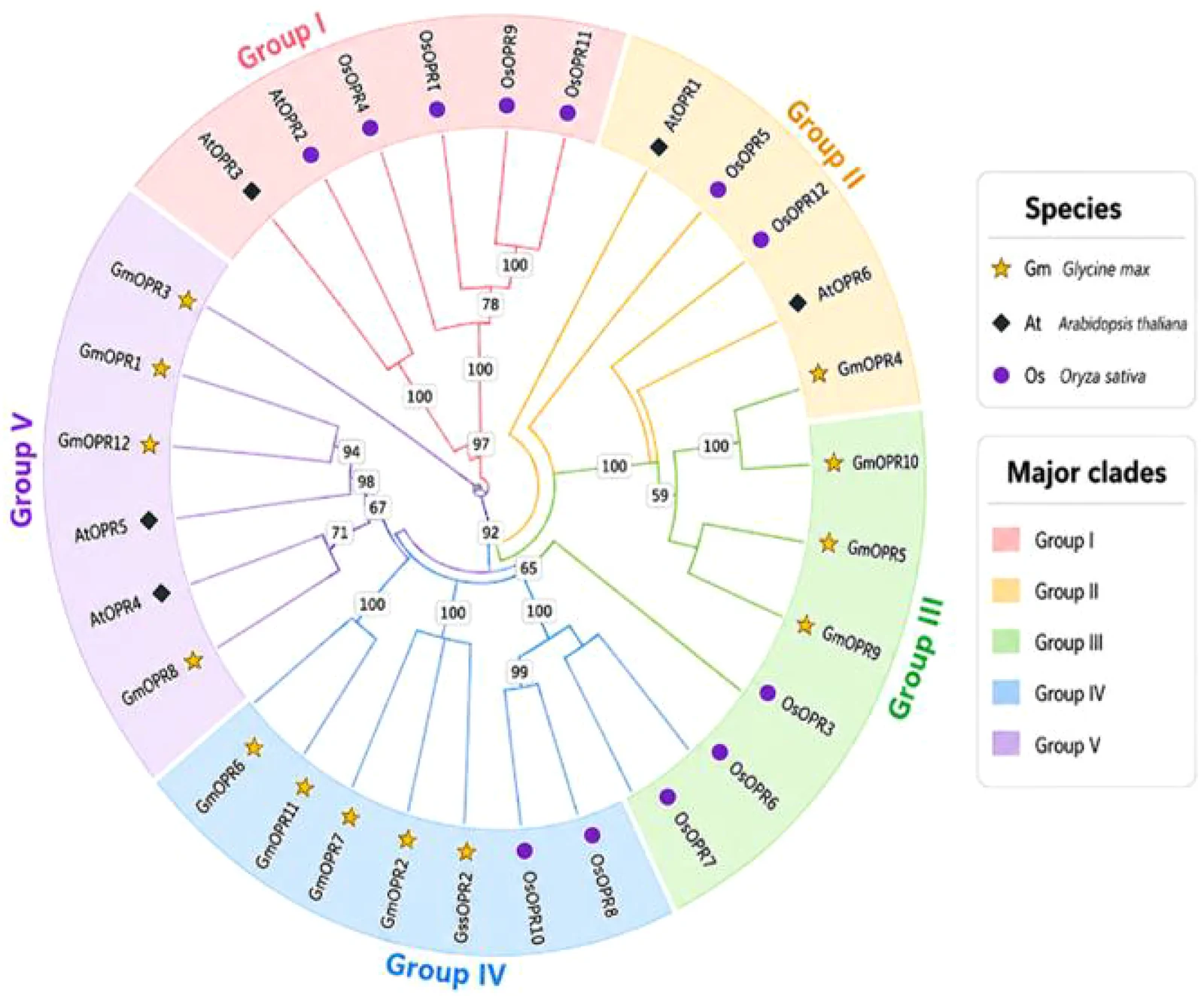
Phylogenetic tree of OPR proteins from Glycine max, Arabidopsis thaliana, and Oryza sativa. The OPR members are divided into five major clades (Groups I–V), indicated by different background colors.

This circular Neighbor-Joining (NJ) phylogenetic tree was constructed using OPR proteins from Glycine max (yellow stars), Arabidopsis thaliana (black diamonds), and Oryza sativa (purple dots), with 1,000 bootstrap replicates to evaluate tree reliability. (**Figure 1**) Numbers at each internal node indicate bootstrap support; higher values reflect greater confidence in branch clustering. All OPR sequences are categorized into five distinct clades (Group I–V), marked with different background colors. Group I (pink) contains OPRs from Arabidopsis and rice only, with no soybean members. Group II (orange) includes OPR genes from Arabidopsis, rice, and soybean GmOPR4. Group III (green) mainly consists of rice OPRs and several soybean OPRs, such as GmOPR5, GmOPR9, and GmOPR10. Group IV (blue) harbors the majority of soybean OPR members, including the target gene GmOPR11, alongside a small number of rice OPR sequences. Group V (purple) is composed of soybean and Arabidopsis OPR genes, with no rice homologs. The overall clustering pattern demonstrates lineage-specific gene duplication and functional divergence of OPR families between monocots (rice) and dicots (soybean and Arabidopsis) during evolution. The 12 soybean OPR genes are unevenly distributed across Groups II, III, IV, and V rather than forming a single clade, which suggests that the soybean OPR family evolved from common ancestral genes and has likely experienced legume-specific segmental duplication events after the divergence of monocots and dicots.

GmOPR4 and GmOPR9 on Gm13/Gm17 appear as a pair in every species with syntenic relationships, confirming that these two genes are products of the soybean whole-genome duplication (WGD). The six syntenic pairs between soybean and Medicago truncatula reveal a legume-specific conserved genomic backbone. The syntenic blocks between soybean and rice OPR genes represent ancient syntenic blocks that trace back to the last common ancestor of angiosperms (predating the monocot–eudicot divergence). Due to the ancient polyploidization event in maize, a single soybean OPR gene detects syntenic signals on both maize subgenomes (**Supplementary Tables 3**–**6**).

**Figure 2:**
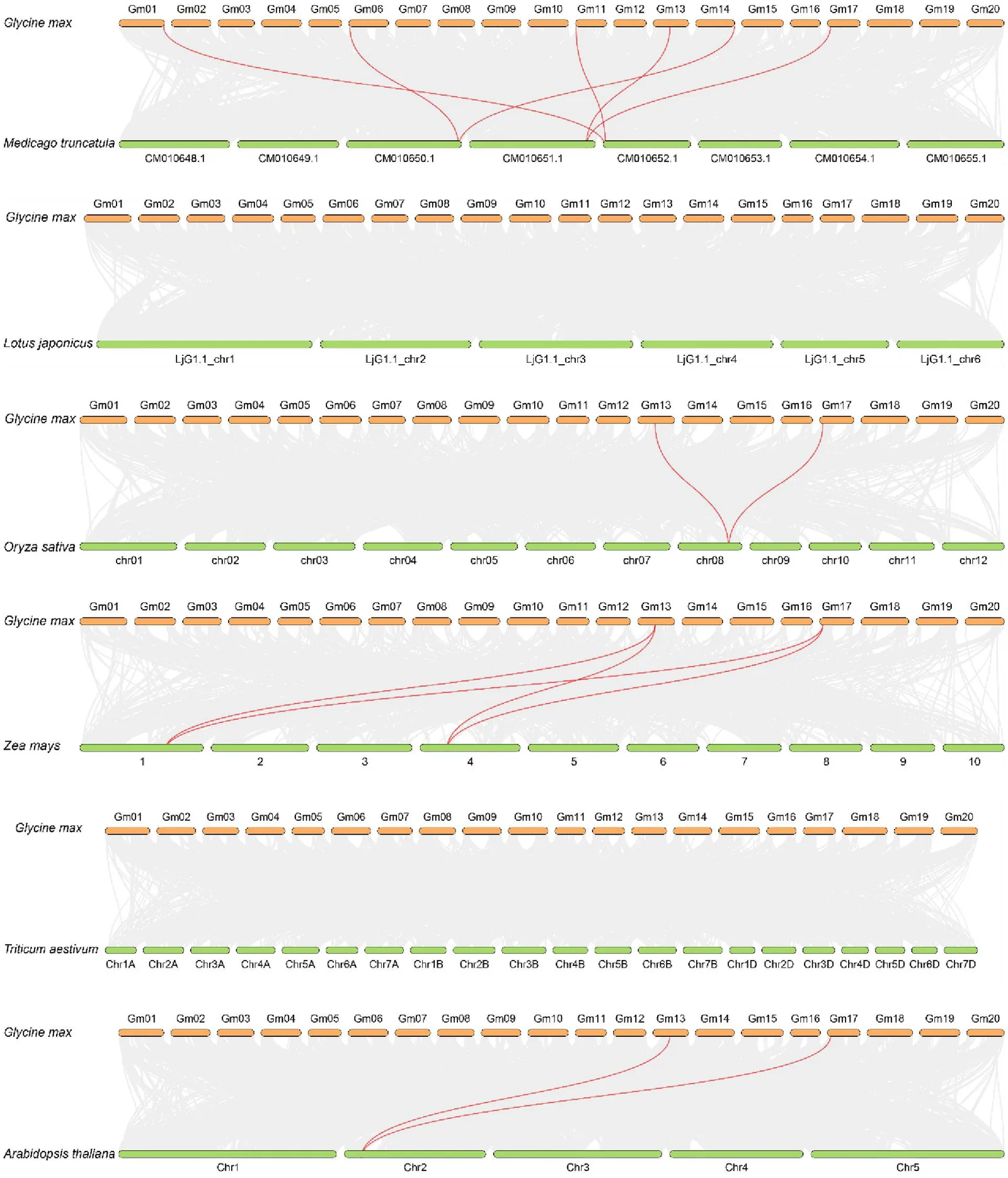
Synteny analysis of *OPR* genes between soybean and other plant species. Synteny relationships of *OPR* genes between soybean (Glycine max) and six plant species (Lotus japonicus, Medicago truncatula, Triticum aestivum, Zea mays, Oryza sativa, and Arabidopsis thaliana) are shown. Orange and green bars represent chromosomes, gray lines indicate collinear blocks, and red lines denote homologous *OPR* gene pairs. More collinear pairs were observed between soybean and Medicago truncatula, fewer with Zea mays, Oryza sativa, and Arabidopsis thaliana, and none with Lotus japonicus or Triticum aestivum, suggesting differential conservation of *OPR* genes across species.

To systematically investigate the evolutionary characteristics of the *OPR* gene family, a comprehensive analysis was performed by integrating phylogenetic analysis with cross-species synteny analysis. The phylogenetic tree revealed that OPR proteins can be classified ino four major evolutionary clades, and synteny analysis further highlighted the differences in evolutionary conservation among these clades, as shown in **Figure 2**. Within legumes, multiple syntenic *OPR* gene pairs were identified between soybean and Medicago, indicating that some members of the legume-specific clade have remained relatively conserved during evolution. However, no obvious syntenic relationships were detected between soybean and Lotus japonicus, suggesting that despite clustering within the same phylogenetic group, these genes may have undergone substantial genomic rearrangements or gene loss events, leading to the absence of detectable synteny. In the conserved angiosperm clade, a number of syntenic *OPR* gene pairs were identified between soybean and rice, maize, as well as Arabidopsis, indicating that these genes likely originated prior to the divergence of monocots and dicots and have been broadly retained during evolution. This observation is consistent with the mixed clustering of OPR proteins from different species in the phylogenetic tree, further supporting their evolutionary conservation. In contrast, although wheat belongs to the Poaceae family and clusters within the Poaceae-specific clade in the phylogenetic tree, no clear syntenic relationships were observed between soybean and wheat. This may be attributed to the complex polyploid nature of the wheat genome and the high frequency of chromosomal rearrangements, which could weaken or obscure syntenic signals between the two species. In summary, the phylogenetic classification and synteny analysis are highly consistent and mutually supportive. On one hand, the conserved angiosperm clade exhibits strong cross-species synteny, reflecting high structural and functional conservation. On the other hand, the legume-specific and Poaceae-specific clades show varying degrees of synteny loss, indicating that the *OPR* gene family has undergone extensive gene duplication, loss, and genomic rearrangements across different plant lineages, thereby driving functional diversification and adaptive evolution.

### *GmOPRs* distribute unevenly across eight soybean chromosomes

**Figure 3:**
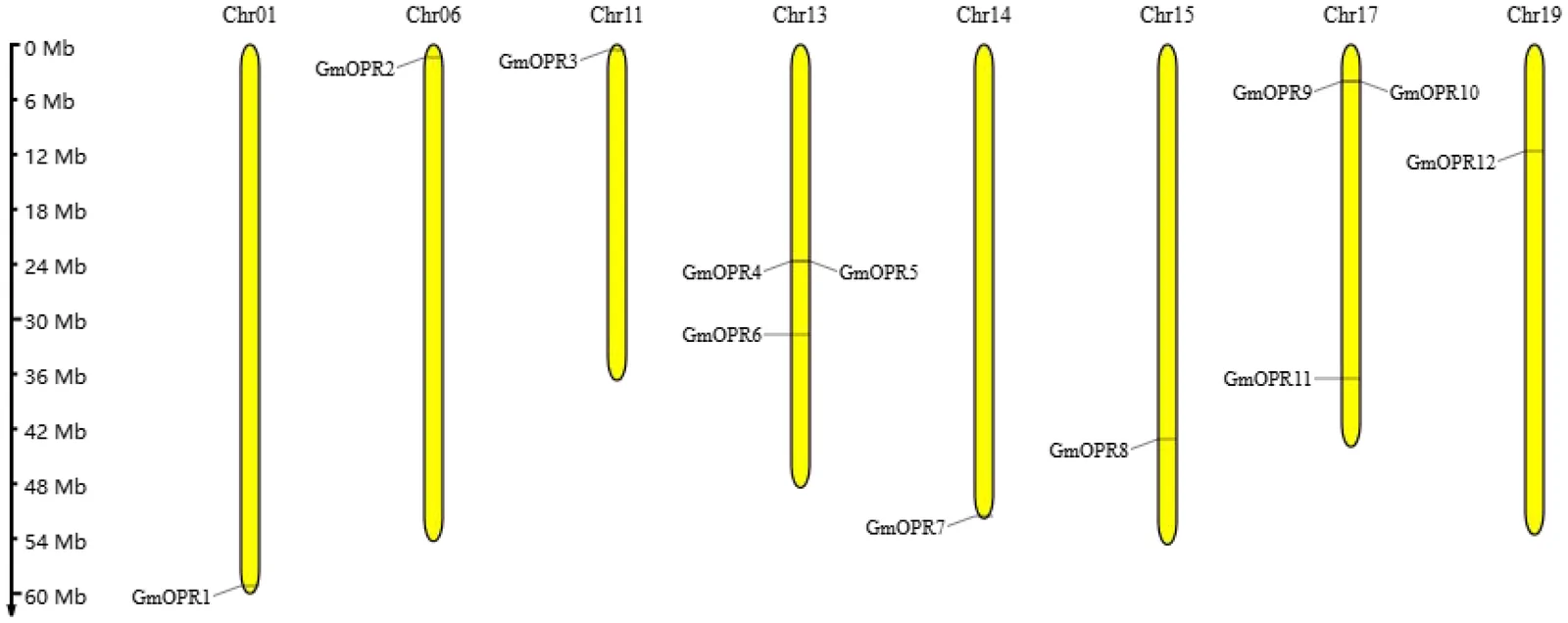
Chromosomal distribution of soybean *OPR* genes. The 12 *GmOPR* genes were mapped to 8 soybean chromosomes (Chr01, Chr06, Chr11, Chr13, Chr14, Chr15, Chr17, and Chr19). The scale bar on the left indicates the chromosome length in megabase pairs (Mb), and gene names are labeled at their corresponding positions.

Regarding the chromosomal distribution of genes (**Figure 3**), *GmOPRs* (*OPR* genes in *Glycine max*) displayed an uneven distribution across eight chromosomes. Specifically, Chr13 harbored three members (*GmOPR4*, *GmOPR5*, *GmOPR6*), and Chr17 contained three members (*GmOPR9*, *GmOPR10*, *GmOPR11*), representing the chromosomes with the highest *GmOPR* gene density. Chr01, Chr06, Chr11, Chr14, Chr15, and Chr19 each carried one *GmOPR* gene. This nonrandom chromosomal distribution pattern of the *GmOPR* gene family may be attributed to segmental duplication or selection pressures during soybean genome evolution.

### Homologous *GmOPRs* share consistent motifs and gene architectures

**Figure 4:**
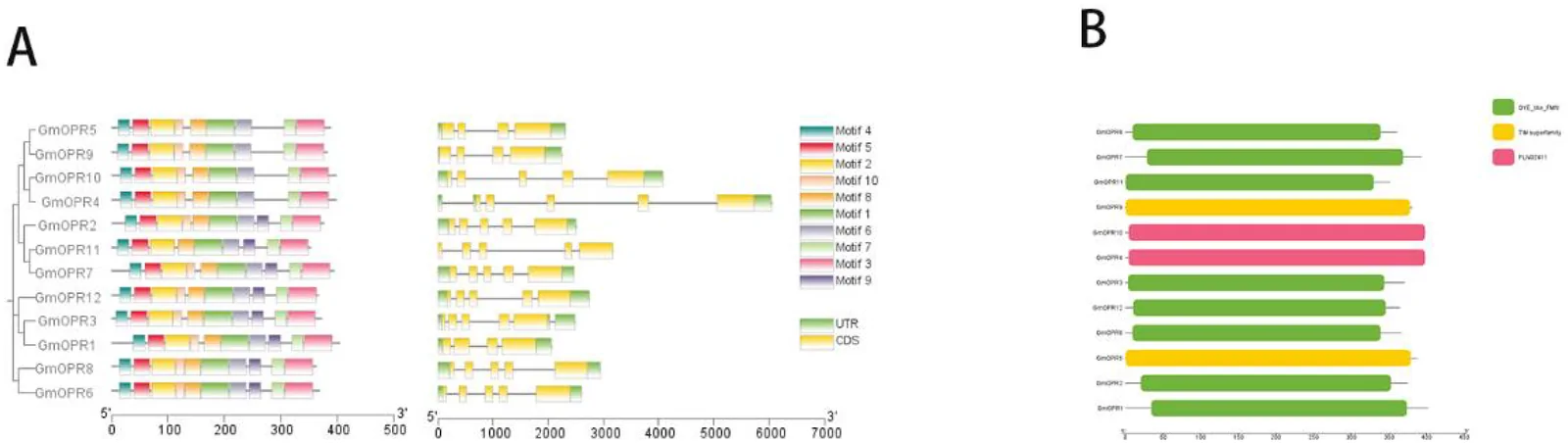
Genomic characterization of the soybean GmOPR family: conserved motifs, gene structures and chromosomal distribution. **(A)** Conserved protein motifs and exon-intron structures of soybean GmOPR genes. The phylogenetic tree on the left corresponds to the clustering classification of the aforementioned NJ tree. Different colored boxes represent ten types of conserved protein motifs; yellow blocks indicate CDS, green blocks represent UTR, and thin black lines stand for introns. **(B)** Chromosomal localization and duplication modes of all 12 soybean GmOPR genes. Green bars denote single-copy loci, yellow bars represent tandem duplicated clusters, and red bars indicate segmental duplicated gene pairs.

The phylogenetic tree on the left corresponds to the clustering classification of the aforementioned NJ tree, and **Figure 4A** displays the conserved protein motifs and exon-intron structures of the 12 soybean *GmOPR* genes. The motif logos of conserved motifs are shown in **Supplementary Figure 1**. Different colored boxes in the motif diagram represent ten categories of conserved protein motifs; yellow blocks correspond to coding sequences (CDS), green blocks denote untranslated regions (UTR), and thin lines between coding regions are introns. All identified GmOPR proteins contain complete core conserved motifs. Genes clustered in the same phylogenetic clade have highly consistent motif arrangement and fragment lengths, which strongly support the reliability of our phylogenetic grouping; only minor length differences are observed among evolutionarily distant genes. Most *GmOPR* genes have a consistent number of exons, whereas *GmOPR4* possesses significantly longer intronic regions, suggesting that intron insertions and deletions act as key drivers of structural divergence during *OPR* family evolution. The target gene *GmOPR11* retains all conserved functional motifs, confirming that it maintains the intact core functional architecture characteristic of the *OPR* family.

**Figure 4B** further illustrates the chromosomal localization and duplication modes of all 12 *GmOPR* genes. Green bars represent single-copy gene loci on chromosomes, yellow bars mark tandem duplicated gene clusters, and red bars indicate segmental duplicated gene pairs. *GmOPR* genes are unevenly distributed across multiple soybean chromosomes. Segmental duplication is identified as the main evolutionary driving force for *OPR* family expansion, with a small number of tandem duplication events acting as supplementary contributors. These two duplication patterns together increase gene copy numbers and explain the scattered distribution of soybean *OPR* homologs across distinct clades in the phylogenetic tree.

Integrated interpretation of phylogenetic clustering, conserved protein motifs, gene structural characteristics, and chromosomal localization yields mutually consistent conclusions. Closely related homologous genes exhibit highly similar motif profiles and exon-intron configurations, and most of these paralogs originate from segmental duplication events. In contrast, evolutionarily distant genes show significant divergence in both gene structure and genomic distribution. Multi-dimensional evidence collectively indicates that the soybean *OPR* family originated from ancient conserved progenitor genes that existed before the divergence of monocots and dicots. Following legume speciation, this gene family underwent lineage-specific expansion mainly through segmental duplication, with tandem duplication playing a secondary role, gradually leading to extensive structural and functional diversification.

### *GmOPR* promoters are enriched with hormone- and stress-responsive cis-elements

**Figure 5:**
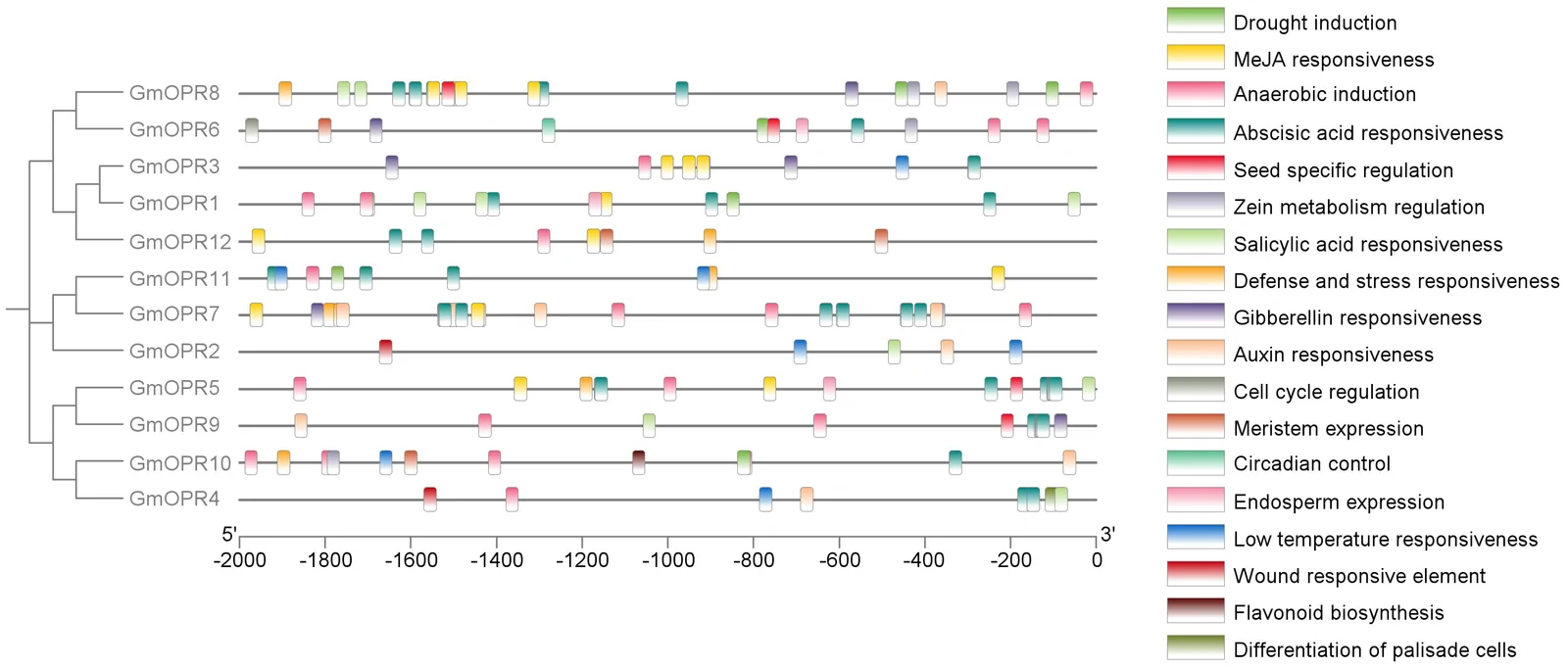
Cis-acting elements of soybean *OPR* genes. Left: Phylogenetic tree constructed from GmOPR protein sequences. Middle: Schematic representation of cis-acting elements in the promoter regions of each gene. Colored boxes represent different types of cis-acting elements, positioned relative to the transcription start site (TSS). The horizontal axis indicates the base pair (bp) distance from the TSS. Right: Legend with functional annotations for each element.

To explore the potential transcriptional regulatory mechanisms and functional divergence of the *GmOPR* gene family, we analyzed the cis-acting elements in the 2000 bp promoter regions upstream of the translation start site of all *GmOPR* genes. Based on the prediction results of promoter *cis*-acting elements (**Figure 5**), the promoter regions of *GmOPR* genes contained a diverse array of *cis*-acting elements, which can be functionally classified into three major categories: elements related to plant growth and development, hormone response elements, and stress response elements. Among the growth and development-related elements, seed-specific regulation elements and endosperm expression elements were frequently detected; seed-specific regulation elements were present in 7 out of 12 *GmOPR* genes, implying a potential role of *GmOPRs* in seed development. For hormone response elements, MeJA responsiveness elements and abscisic acid responsiveness elements were dominant—MeJA responsiveness elements were identified in 9 *GmOPR* genes, while abscisic acid responsiveness elements were found in 8 members, suggesting that *GmOPR* gene expression may be modulated by MeJA and ABA signaling pathways to adapt to developmental and stress conditions. In terms of stress response elements, defense and stress responsiveness elements and low temperature responsiveness elements were abundant; defense and stress responsiveness elements were detected in all *GmOPR* genes except *GmOPR3* and *GmOPR6*, and low temperature responsiveness elements were present in 6 *GmOPR* genes. Notably, *GmOPR8* contained a unique combination of multiple stress-responsive elements (drought induction, MeJA responsiveness, ABA responsiveness among others), indicating that this member may play a pivotal role in soybean’s integrated response to multiple abiotic and biotic stresses. Overall, the *cis*-acting elements within the *GmOPR* gene family showed no uniform distribution pattern, with variations in element types and copy numbers among different *GmOPR* members—this diversity may underlie the functional specialization of *GmOPR*s in regulating distinct biological processes in soybean.

Hormone-responsive elements are dominant, accounting for 102 (62.2%) of all cis-elements, with ABA-responsive elements (47) and MeJA-responsive elements (30) being the most abundant — this is highly consistent with the core functions of OPR genes in jasmonic acid (JA) biosynthesis and ABA signaling pathways. The promoters of GmOPR7 and GmOPR8 are the most complex, each containing 26 cis-elements with dense clusters of hormone- and stress-responsive elements, suggesting that these two genes may be subject to synergistic regulation by multiple signals. The GmOPR2 promoter is the simplest, harboring only 5 elements, implying a relatively restricted regulatory mode that may involve constitutive or tissue-specific expression. Stress-responsive elements are broadly distributed, with anaerobic induction elements present in the promoters of 11 out of 12 genes, indicating that the OPR gene family may be widely involved in the hypoxic stress response. Sheet 3 further provides the precise coordinates of 81 element loci (e.g., ABA-responsive elements in the GmOPR1 promoter are located at 578–608 bp, 1088–1118 bp, and 1737–1767 bp) (**Supplementary Tables 7**-**9**).

### *GmOPRs* exhibit distinct tissue-specific expression patterns

**Figure 6:**
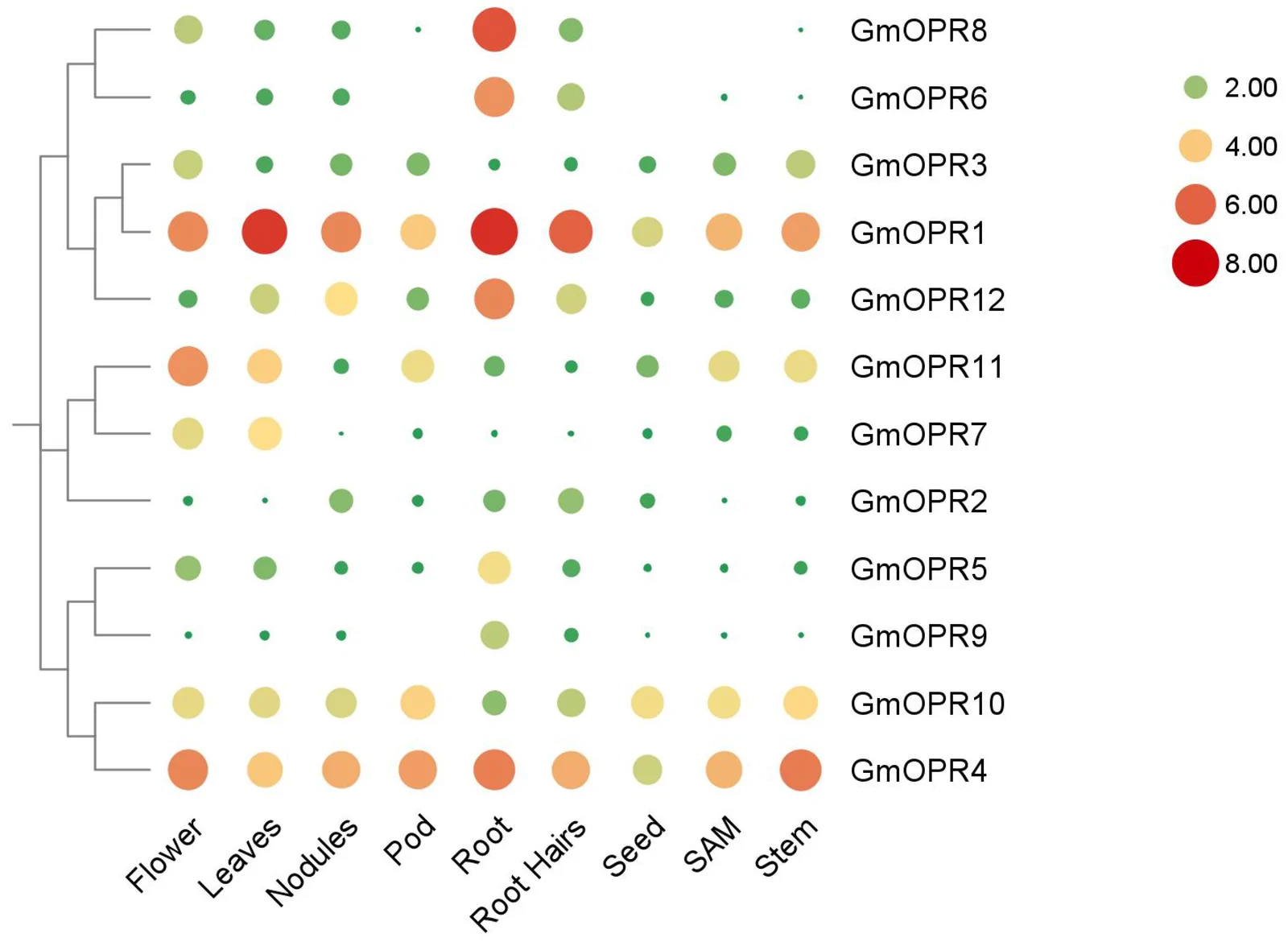
Tissue expression profiles of the *GmOPR* gene family during soybean growth and development. This bubble heatmap was generated based on FPKM-normalized expression data of 9 soybean tissues downloaded from the JGI Phytozome database. The x-axis represents different tissues and organs, including flower, leaves, nodules, pod, root, root hairs, seed, shoot apical meristem (SAM) and stem; the y-axis represents the 12 identified *GmOPR* genes. The size and color depth of each circle correspond to the expression level of the corresponding gene in a given tissue. The hierarchical clustering tree on the left was constructed according to the similarity of tissue expression patterns among *GmOPR* genes.

To clarify the tissue-specific expression characteristics of the soybean *GmOPR* gene family, we first analyzed the expression patterns of 12 *GmOPR* genes in nine different tissues using transcriptome data from the Phytozome database (**Figure 6**). The results revealed distinct tissue-specific expression patterns among the *GmOPR* family members: most members (e.g., *GmOPR1*, *GmOPR4*, *GmOPR6*, *GmOPR8*, and *GmOPR12*) were significantly upregulated in root tissue, with *GmOPR1* and *GmOPR8* showing the highest expression levels, suggesting that they may play key roles in soybean root development or stress responses; *GmOPR1*, *GmOPR4*, and *GmOPR10* were highly expressed in all tested tissues, implying their involvement in regulating fundamental cellular metabolic processes; some members exhibited obvious tissue preference, such as *GmOPR11*, which was highly expressed in flowers, leaves, pods, shoot apical meristem (SAM), and stems; *GmOPR7*, which was mainly expressed in flowers and leaves; while *GmOPR1,GmOPR4,GmOPR6,GmOPR8* and *GmOPR12* showed root-specific high expression.

**Figure 7:**
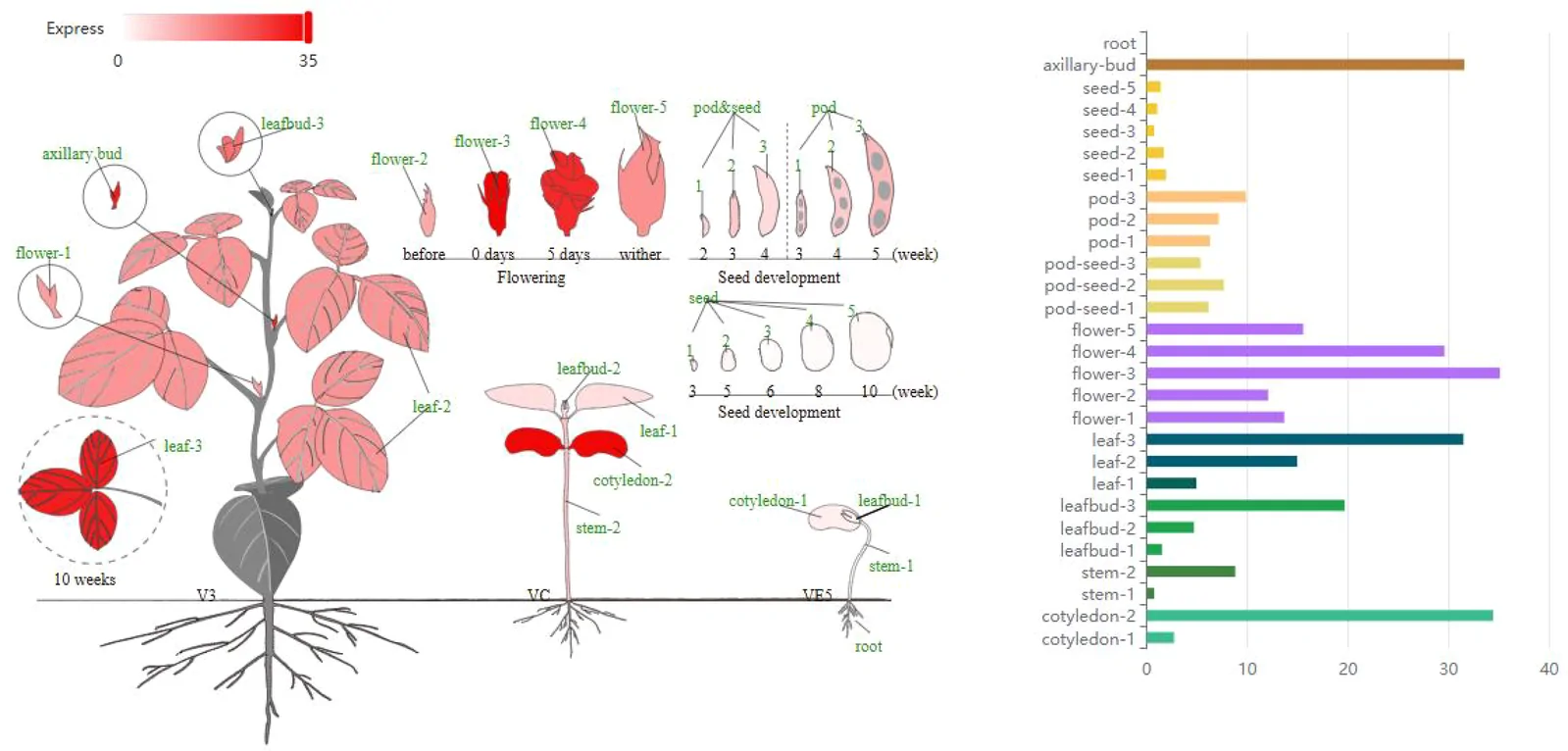
Spatiotemporal expression pattern of *GmOPR*. The expression levels of *OPR* in soybean tissues at different developmental stages are shown (left), with darker colors representing higher expression levels. The abscissa indicates the expression level, and the ordinate represents samples from different tissues at various developmental stages (right).

Previously, through transcriptome analysis of the two parents, we identified differentially expressed genes and their metabolic pathways. Combined with SLAF sequencing, we identified a novel deterioration-resistant gene, Glyma.17G209900 (*GmOPR11*), as a candidate gene for seed deterioration resistance. To further verify and refine the spatiotemporal expression pattern of this key member, *GmOPR11* was selected for expression analysis at different developmental stages and in different tissues (**Figure 7**). The results showed that the expression of *GmOPR11* exhibited significant spatiotemporal specificity: in reproductive organs, its expression level gradually increased with flower development, peaked at the flowering stage (flower-3), and remained high during the early stages of pod and seed development; in vegetative organs, it was highly expressed in cotyledons (cotyledon-2), axillary buds, and mature leaves (leaf-3), while its expression level was extremely low in roots, stems, and other tissues. These results are highly consistent with the high expression characteristics of *GmOPR11* in flowers, leaves, and pods shown in **Figure 6**, further confirming the expression specificity of this gene and suggesting that it may play an important role in soybean flower organ development, axillary bud growth, and functional maintenance of mature leaves.

Taken together, the results from **Figure 6** and **7** show that the expression patterns of different members of the *GmOPR* family are significantly different, reflecting their potential division of labor in the development and functional regulation of different soybean organs. Among them, the spatiotemporal expression characteristics of *GmOPR11* are particularly prominent, making it an important candidate gene for subsequent functional research.

### *GmOPR11* expression negatively correlates with seed deterioration resistance

**Figure 8:**
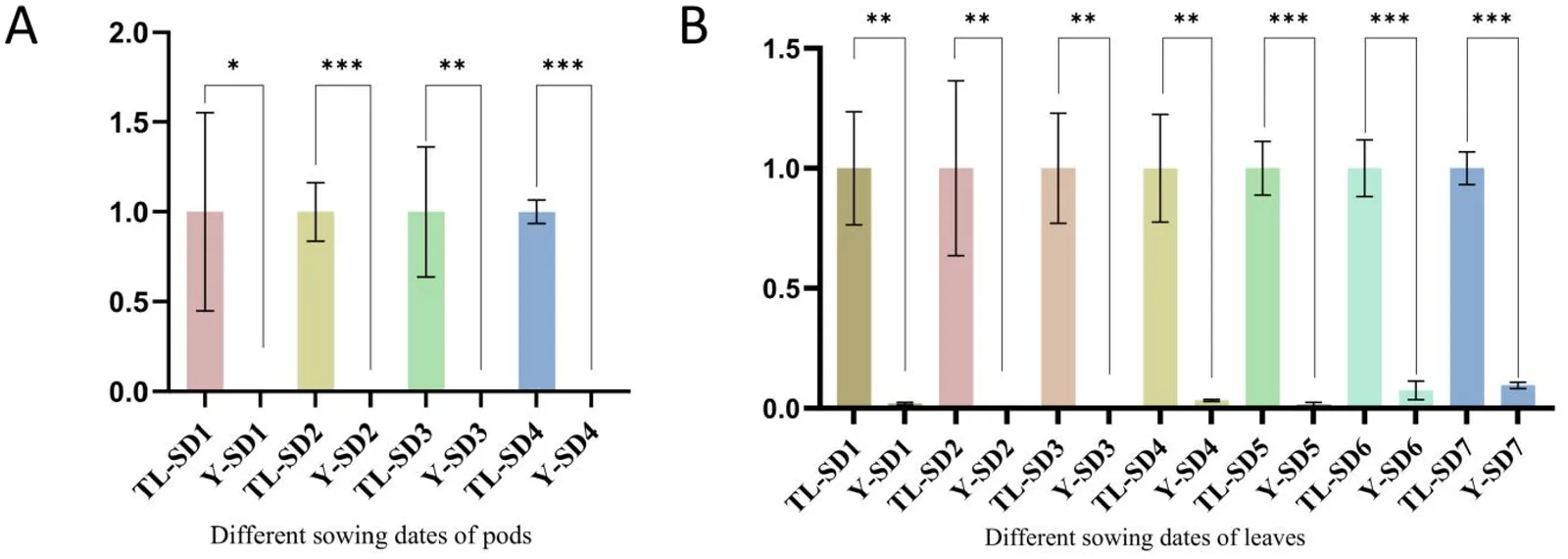
Expression levels of GmOPR11 in different tissues of the deterioration-resistant soybean line (Y17166) and the deterioration-susceptible line (Tianlong No.1) under different sowing dates(SD). The X-axis represents seven sowing dates, numbered 1 to 7; the Y-axis indicates the relative gene expression levels. The deterioration-susceptible line KF1 was used as the control for the resistant line Y17166. **(A)** Expression levels of the *GmOPR11* gene in pods. **(B)** Expression levels of the *GmOPR11* gene in leaves. Samples from sowing dates 5 to 7 had not yet podded at the sampling time. * P < 0.05; ** P < 0.01; *** P < 0.001.

In previous studies, we identified a candidate gene for seed deterioration resistance, *GmOPR11*, through combined analysis. Therefore, we investigated its tissue-specific expression levels in leaves and pods sampled uniformly at the early pod-setting stage (R3 stage) from deterioration-resistant and deterioration-sensitive soybean varieties under different sowing dates (**Figure 8**). As shown in **Figures 8A, B**, *GmOPR11* exhibited relatively high expression levels in the deterioration-sensitive parent, while its expression was extremely low or even undetectable in the deterioration-resistant parent. Notably, no normal pod samples were obtained for the SD5 to SD7 treatments due to pod formation failure, and only leaf samples were analyzed for these three groups. These results indicate a negative correlation between the expression level of *GmOPR11* and soybean deterioration resistance.

## Discussion

*OPR*s constitute a multi-gene family. Previous studies have analyzed the structural, evolutionary and functional characteristics of *OPR*s in *Arabidopsis thaliana*, rice (Mekkaoui et al., 2025; Li et al., 2011). Zhang et al. identified 8 *OPR* genes in maize, and subsequent analysis demonstrated that these genes are localized in the cytoplasm or peroxisomes, and undergo specific regulation in response to stress-related hormones, wounding or pathogen infection (Zhang et al., 2005). Li et al. characterized 13 *OPR* genes in rice, whose functions are associated with hormone regulation, wounding as well as other abiotic and biotic stresses (Li et al., 2011). Mou et al. identified 14 *OPR* genes in wheat, which are primarily involved in growth and development, hormone signaling, and responses to biotic and abiotic stresses (Mou et al., 2019). In soybean, the expression patterns of *GmOPR*s are similar to those of *OPR*s in Arabidopsis thaliana and wheat, with predominant expression in roots and strong tissue specificity (Biesgen and Weiler, 1999; Mou et al., 2019).

Comparing our results with known *OPR* functions in Arabidopsis, rice, maize, and wheat helps to understand the conservatism and species specificity of *OPRs* in regulating seed vigor. In Arabidopsis, *OPR3* is the main enzyme for JA biosynthesis, and its mutant is sensitive to photostress and osmotic stress (Cheng et al., 2023), indicating that the OPR-JA axis plays a conserved role in seed stress responses. In rice, *OsOPR7* has a similar function (Asfaw et al., 2022). In maize, *OPR7* and *OPR8* are responsible for JA synthesis (He et al., 2020) and are essential for reproductive development and stress defense (Yan et al., 2014). Overexpression of *AtOPR3* in wheat enhances stress tolerance (Pigolev et al., 2023), but also delays seed germination (Pigolev et al., 2018). In contrast, loss-of-function of the *OPR-III* gene cluster leads to elongated roots (Gabay et al., 2023), suggesting that JA dosage is critical for seed development.

Consistent with the expression data of GmOPR11 presented in **Figures 6**–**8**, this study is the first to establish a direct connection between GmOPR11 and soybean seed storage deterioration, revealing that high transcript abundance of this gene is negatively correlated with seed deterioration resistance. This unique observation differs from the conserved positive function of OPRs in sustaining seed vigor reported in Arabidopsis and rice. We propose that this functional divergence can be explained by the JA dosage effect: moderate JA accumulation facilitates seed maturation and cell wall strengthening, while excessive JA disturbs endogenous hormone homeostasis and triggers massive ROS bursts, consequently accelerating lipid peroxidation and seed aging.

However, a large body of research has demonstrated that *OPR* genes are associated with diverse biotic and abiotic stresses (Chen et al., 2026; Tian et al., 2020; Wasternack et al., 2006). *GmOPR* encodes 12-oxophytodienoate reductase (*OPR*), which acts as a key rate-limiting enzyme in the jasmonic acid (JA) biosynthetic pathway (Turner et al., 2002; Wang et al., 2022; Howe et al., 2018). Consistent with the qRT-PCR data shown in **Figure 8**, the transcript abundance of GmOPR11 was markedly higher in the deterioration-susceptible soybean cultivar than in the resistant counterpart. This negative correlation between *GmOPR11* expression and seed deterioration resistance may be ascribed to the dual roles of jasmonic acid (JA) in regulating seed physiological processes: on the one hand, moderate JA accumulation during seed maturation facilitates nutrient deposition and cell wall fortification (Ghassemi-Golezani et al., 2018), thereby improving seed storage stability; on the other hand, excessive JA biosynthesis (driven by high-level *OPR* gene expression) is likely to perturb the equilibrium of the endogenous hormone network (Hazman et al., 2015) or trigger excessive reactive oxygen species (ROS) accumulation (Dong et al., 2013). This, in turn, accelerates seed aging, induces lipid peroxidation, and impairs seed viability during storage—all of which are hallmark features of seed deterioration.

Phylogenetic analysis based on **Figure 1** revealed that OPR proteins from *Glycine max, Arabidopsis thaliana*, and *Oryza sativa* were divided into five subgroups (Groups I–V), which could be further integrated into four major evolutionary clades: conserved angiosperm clade (Group I), conserved dicot clade (Group II), Poaceae-specific clade (Groups III and IV), and legume-Brassicaceae shared clade (Group V harboring *AtOPR4*). Group I only contained OPR sequences from Arabidopsis and rice without soybean homologs, so it is not a legume-specific branch. This classification is generally consistent with previous studies on rice and wheat (Li et al., 2011; Mou et al., 2019), which also observed lineage-specific clustering of OPR proteins. Compared with prior single- or dual-species phylogenetic analyses, the three-species dataset in this study constructs a clearer evolutionary framework by covering a legume crop (soybean), a dicot model (Arabidopsis), and a monocot model (rice). The 12 soybean *GmOPR* genes are scattered across Group II, III, IV and V rather than forming an independent legume-specific clade, which indicates that the expansion of the soybean *OPR* family was jointly driven by ancient conserved ancestral genes and recent lineage-specific gene duplication events after the divergence of major plant lineages. Similar lineage-specific expansion patterns of *OPRs* have also been reported in wheat (Mou et al., 2019) and maize (Li et al., 2023).

Promoter cis-element analysis (**Figure 5**) confirmed that the upstream sequences of all GmOPR genes are enriched with MeJA- and ABA-responsive cis-elements, as well as abundant stress-related and seed development-related regulatory motifs. Transcription factors function as molecular hubs that integrate biotic and abiotic stress signals and mediate crosstalk among hormone signaling pathways (Liu et al., 2024). The coexistence of multiple types of cis-elements in *GmOPR* promoters provides a transcriptional basis for the involvement of *OPR* in multiple pathways, including seed development and oxidative aging. Tissue expression profiling shown in **Figure 6** revealed that most *GmOPR* genes are highly expressed in roots, a pattern consistent with the *OPR* expression profiles in Arabidopsis and wheat (Mou et al., 2019), suggesting that the JA signaling pathway in roots is highly conserved across both dicots and monocots. In contrast, *GmOPR11* displayed a unique reproductive organ-biased expression profile in **Figure 6** and **7**: it accumulates high transcript levels in flowers, pods and mature leaves, while showing relatively low expression in roots and stems. This distinct expression signature implies that this paralog has evolved a specialized function linked to seed deterioration, which differs from other members of the *GmOPR* family.

In this study, seven sowing date gradients were used as field experimental materials, and qRT-PCR detection based on **Figure 8** confirmed that the expression level of GmOPR11 was significantly negatively correlated with soybean seed deterioration resistance. Under all treatments, the deterioration-susceptible cultivar Tianlong No.1 showed substantially higher *GmOPR11* transcript abundance in various tissues than the deterioration-resistant line Y17166. For the late-sown groups SD5–SD7, which failed to form normal pods due to photothermal imbalance, leaf expression data still maintained the same differential trend. This negative correlation can be fully explained by the dual regulatory effects of JA: moderate JA accumulation during mid-to-late seed development promotes nutrient deposition and cell wall reinforcement, thereby enhancing storage vigor (Sood, 2023); however, high expression of *GmOPR11* continuously activates the JA biosynthesis pathway, and excessive JA disrupts endogenous hormone homeostasis and induces massive accumulation of reactive oxygen species, which in turn triggers lipid peroxidation, protein denaturation, and DNA damage, ultimately accelerating post-harvest seed deterioration and reducing germination rate and seedling vigor in soybean (Dong et al., 2013; Hazman et al., 2015). Most existing studies have focused on the roles of OPR in disease resistance, salt tolerance, and cold tolerance. This study is the first to associate the soybean OPR-JA pathway with seed storage deterioration, thereby expanding the research boundary of JA in pos-harvest quality regulation in crops.

In summary, this study provides the first systematic characterization of the OPR gene family in soybean, revealing lineage-specific evolutionary patterns, structural diversity, and tissue-specific expression profiles. The identification of *GmOPR11* as a candidate gene negatively associated with seed deterioration resistance offers a new perspective on the role of JA metabolism in seed quality. While the precise molecular mechanisms remain to be elucidated, our findings lay a foundation for future functional studies and breeding efforts aimed at improving soybean seed storage performance.

## Conclusions

This study identified 12 *GmOPR* genes in soybean, unevenly distributed across 8 chromosomes. These genes exhibit diverse physicochemical properties, most being acidic and hydrophilic, with subcellular localization in mitochondria, cytoplasm, or chloroplasts. Phylogenetic analysis classified the *OPR* genes from soybean, rice, Arabidopsis, alfalfa, lotus, maize and wheat into four clades, indicating that the *OPR* gene family has undergone varying degrees of functional differentiation and adaptive evolution among different plant lineages. *Cis*-acting elements, gene structures, and motif compositions reflect functional specialization. Tissue-specific expression revealed predominant root expression and distinct patterns among members. Notably, *GmOPR11* expression negatively correlates with soybean deterioration resistance, potentially due to excessive jasmonic acid accumulation disrupting hormone balance or inducing reactive oxygen species. This systematic characterization provides a foundation for exploring *GmOPR* functions and developing soybean cultivars with improved storage stability.

## Data Availability

The original contributions presented in the study are included in the article/**Supplementary Material**. Further inquiries can be directed to the corresponding authors.

## References

[B1] Al Loman A. Ju L. K. (2017). Enzyme-based processing of soybean carbohydrate: Recent developments and future prospects. Enzyme Microb. Technol. 106, 35–47. doi: 10.1016/j.enzmictec.2017.06.013 28859808

[B2] Asfaw K. G. Liu Q. Eghbalian R. Purper S. Akaberi S. Dhakarey R. . (2022). The jasmonate biosynthesis gene OsOPR7 can mitigate salinity induced mitochondrial oxidative stress. Plant Sci. 316, 111156. doi: 10.1016/j.plantsci.2021.111156 35151439

[B3] Baributsa D. Baoua I. B. (2022). Hermetic bags maintain soybean seed quality under high relative humidity environments. J. Stored Prod. Res. 96, 101952. doi: 10.1016/j.jspr.2022.101952 35340696 PMC8935379

[B4] Biesgen C. Weiler E. (1999). Structure and regulation of OPR1 and OPR2, two closely related genes encoding 12-oxophytodienoic acid-10, 11-reductases from Arabidopsis thaliana. Planta 208, 155–165. doi: 10.1007/s004250050545 10333582

[B5] Chen Z. Chen E. Zhang J. Ying Z. (2026). Jasmonic acid enhances thermotolerance in hybrid Pennisetum via activation of the α-linolenic acid metabolism pathway. Front. Plant Sci. 17, 1756886. doi: 10.3389/fpls.2026.1756886 42273213 PMC13246406

[B6] Cheng H. Hao M. Sang S. Wen Y. Cai Y. Wang H. . (2023). Establishment of new convenient two-line system for hybrid production by targeting mutation of OPR3 in allopolyploid Brassica napus. Hortic. Res. 10, uhad218. doi: 10.1093/hr/uhad218 38077491 PMC10699839

[B7] Dong W. Wang M. Xu F. Quan T. Peng K. Xiao L. . (2013). Wheat oxophytodienoate reductase gene TaOPR1 confers salinity tolerance via enhancement of abscisic acid signaling and reactive oxygen species scavenging. Plant Physiol. 161, 1217–1228. doi: 10.1104/pp.112.211854 23321418 PMC3585591

[B8] Elhalis H. Chin X. H. Chow Y. (2024). Soybean fermentation: Microbial ecology and starter culture technology. Crit. Rev. Food Sci. Nutr. 64, 7648–7670. doi: 10.1080/10408398.2023.2188951 36916137

[B9] Fleming A. M. Zhu J. Ding Y. Burrows C. J. (2019). Location dependence of the transcriptional response of a potential G-quadruplex in gene promoters under oxidative stress. Nucleic Acids Res. 47, 5049–5060. doi: 10.1093/nar/gkz207 30916339 PMC6547423

[B10] Gabay G. Wang H. Zhang J. Moriconi J. I. Burguener G. F. Gualano L. D. . (2023). Dosage differences in 12-OXOPHYTODIENOATE REDUCTASE genes modulate wheat root growth. Nat. Commun. 14, 539. doi: 10.1038/s41467-023-36248-y 36725858 PMC9892559

[B11] Ghassemi-Golezani K. Farhangi-Abriz S. Bandehagh A. (2018). Salicylic acid and jasmonic acid alter physiological performance, assimilate mobilization and seed filling of soybean under salt stress. Acta Agriculturae Slovenica 111, 597–607. doi: 10.14720/aas.2018.111.3.08

[B12] Guo B. Sun L. Jiang S. Ren H. Sun R. Wei Z. . (2022). Soybean genetic resources contributing to sustainable protein production. Theor. Appl. Genet. 135, 4095–4121. doi: 10.1007/s00122-022-04222-9 36239765 PMC9561314

[B13] Hazman M. Hause B. Eiche E. Nick P. Riemann M. (2015). Increased tolerance to salt stress in OPDA-deficient rice ALLENE OXIDE CYCLASE mutants is linked to an increased ROS-scavenging activity. J. Exp. Bot. 66, 3339–3352. doi: 10.1093/jxb/erv142 25873666 PMC4449546

[B14] He Y. Borrego E. J. Gorman Z. Huang P.-C. Kolomiets M. V. (2020). Relative contribution of LOX10, green leaf volatiles and JA to wound-induced local and systemic oxylipin and hormone signature in Zea mays (maize). Phytochemistry 174, 112334. doi: 10.1016/j.phytochem.2020.112334 32172019

[B15] Howe G. A. Major I. T. Koo A. J. (2018). Modularity in jasmonate signaling for multistress resilience. Annu. Rev. Plant Biol. 69, 387–415. doi: 10.1146/annurev-arplant-042817-040047 29539269

[B16] Kafer J. M. Molinari M. D. C. Henning F. A. Alcantara L. V. S. Nepomuceno A. L. Mertz-Henning L. M. (2025). Transcriptomic insights into soybean seed deterioration in uncontrolled storage. J. Seed Sci. 47, e202547002. doi: 10.1590/2317-1545v47289243 41099703

[B17] Li W. Li H. Liu Y. Gong X. Wei S. Gu S. (2023). Pan-genomic identification and expression pattern analysis of the OPR gene family in maize. Acta Agriculturae Boreali-Sinica. 38, 51–59.

[B18] Li W. Zhou F. Liu B. Feng D. He Y. Qi K. . (2011). Comparative characterization, expression pattern and function analysis of the 12-oxo-phytodienoic acid reductase gene family in rice. Plant Cell Rep. 30, 981–995. doi: 10.1007/s00299-011-1002-5 21249367

[B19] Lin Y. Xu H. Yin G. Zhou Y. Lu X. Xin X. (2022). Dynamic changes in membrane lipid metabolism and antioxidant defense during soybean (Glycine max L. Merr.) seed aging. Front. Plant Sci. 13, 908949. doi: 10.3389/fpls.2022.908949 35812982 PMC9263854

[B20] Liu S. Sun R. Zhang X. Feng Z. Wei F. Zhao L. . (2020). Genome-wide analysis of OPR family genes in cotton identified a role for GhOPR9 in Verticillium dahliae resistance. Genes 11, 1134. doi: 10.3390/genes11101134 32992523 PMC7600627

[B21] Liu F. Xi M. Liu T. Wu X. Ju L. Wang D. (2024). The central role of transcription factors in bridging biotic and abiotic stress responses for plants ' resilience. New Crops 1, 100005. doi: 10.1016/j.ncrops.2023.11.003 38826717

[B22] Mekkaoui K. Baral R. Smith F. Klein M. Feussner I. Hause B. (2025). Transcriptomics and trans-organellar complementation reveal limited signaling of 12-cis-oxo-phytodienoic acid during early wound response in Arabidopsis. Nat. Commun. 16, 5218. doi: 10.1038/s41467-025-61832-9 40691436 PMC12280005

[B23] Min C. W. Lee S. H. Cheon Y. E. Han W. Y. Ko J. M. Kang H. W. . (2017). In-depth proteomic analysis of Glycine max seeds during controlled deterioration treatment reveals a shift in seed metabolism. J. Proteomics 169, 125–135. doi: 10.1016/j.jprot.2017.06.022 28669816

[B24] Mou Y. Liu Y. Tian S. Guo Q. Wang C. Wen S. (2019). Genome-wide identification and characterization of the OPR gene family in wheat (Triticum aestivum L.). Int. J. Mol. Sci. 20, 1914. doi: 10.3390/ijms20081914 31003470 PMC6514991

[B25] Pigolev A. V. Miroshnichenko D. N. Dolgov S. V. Alekseeva V. V. Pushin A. S. Degtyaryova V. I. (2023). Endogenously produced jasmonates affect leaf growth and improve osmotic stress tolerance in emmer wheat. Biomolecules 13, 1775. doi: 10.3390/biom13121775 38136646 PMC10742046

[B26] Pigolev A. V. Miroshnichenko D. N. Pushin A. S. Terentyev V. V. Boutanayev A. M. Dolgov S. V. . (2018). Overexpression of Arabidopsis OPR3 in hexaploid wheat (Triticum aestivum L.) alters plant development and freezing tolerance. Int. J. Mol. Sci. 19, 3989. doi: 10.3390/ijms19123989 30544968 PMC6320827

[B27] Pinheiro D. T. Dias D. C. F. S. Medeiros A. D. Ribeiro J. P. O. Silva F. L. Silva L. J. (2021). Weathering deterioration in pre-harvest of soybean seeds: physiological, physical, and morpho-anatomical changes. Sci. Agric. 78, e20200166. doi: 10.1590/1678-992x-2020-0166 41099703

[B28] Rahman M. M. Mat K. Ishigaki G. Akashi R. (2021). A review of okara (soybean curd residue) utilization as animal feed: nutritive value and animal performance aspects. Anim. Sci. J. 92, e13594. doi: 10.1111/asj.13594 34289204

[B29] Rao P. J. M. Pallavi M. Bharathi Y. Priya P. B. Sujatha P. Prabhavathi K. (2023). Insights into mechanisms of seed longevity in soybean: a review. Front. Plant Sci. 14, 1206318. doi: 10.3389/fpls.2023.1206318 37546268 PMC10400919

[B30] Ruan J. Zhou Y. Zhou M. Yan J. Khurshid M. Weng W. . (2019). Jasmonic acid signaling pathway in plants. Int. J. Mol. Sci. 20, 2479. doi: 10.3390/ijms20102479 31137463 PMC6566436

[B31] Schaller F. Biesgen C. Müssig C. Altmann T. Weiler E. W. (2000). 12-Oxophytodienoate reductase 3 (OPR3) is the isoenzyme involved in jasmonate biosynthesis. Planta 210, 979–984. doi: 10.1007/s004250050706 10872231

[B32] Sood M. (2023). Jasmonates: “The master switch” for regulation of developmental and stress responses in plants. J. Plant Growth Regul. 42, 5247–5265. doi: 10.1007/s00344-023-11047-3 30311153

[B33] Tian X. Wang F. Zhao Y. Lan T. Yu K. Zhang L. . (2020). Heat shock transcription factor A1b regulates heat tolerance in wheat and Arabidopsis through OPR3 and jasmonate signalling pathway. Plant Biotechnol. J. 18, 1109. doi: 10.1111/pbi.13268 31559685 PMC7152600

[B34] Turner J. G. Ellis C. Devoto A. (2002). The jasmonate signal pathway. Plant Cell 14, S153–S164. doi: 10.1105/tpc.000679 12045275 PMC151253

[B35] Wang Y. Wang M. Wang J. Zhang Q. Xie L. (2022). Genome-wide identification and expression analysis of soybean OPR gene family. Soybean Sci. 41, 129–139. doi: 10.1016/j.jplph.2005.10.014 37299081

[B36] Wasternack C. Stenzel I. Hause B. Hause G. Kutter C. Maucher H. . (2006). The wound response in tomato–role of jasmonic acid. J. Plant Physiol. 163, 297–306. doi: 10.1016/j.jplph.2005.10.014 16368162

[B37] Yan Y. Huang P. C. Borrego E. Kolomiets M. (2014). New perspectives into jasmonate roles in maize. Plant Signaling Behav. 9, e970442. doi: 10.4161/15592316.2014.970442 25482807 PMC4623489

[B38] Yin G. Whelan J. Wu S. Zhou J. Chen B. Chen X. . (2016). Comprehensive mitochondrial metabolic shift during the critical node of seed ageing in rice. PloS One 11, e0148013. doi: 10.1371/journal.pone.0148013 27124767 PMC4849721

[B39] Zhang J. Simmons C. Yalpani N. Crane V. Wilkinson H. Kolomiets M. (2005). Genomic analysis of the 12-oxo-phytodienoic acid reductase gene family of Zea mays. Plant Mol. Biol. 59, 323–343. doi: 10.1007/s11103-005-8883-z 16247560

