# Supplementary material for: The OPR gene family: bioinformatics characterization and functional implications for soybean seed deterioration
Source: Front Plant Sci. 2026 Aug 11;17:1880971. doi: 10.3389/fpls.2026.1880971 (PMC13503528; doi:10.3389/fpls.2026.1880971)
Supplement: Supplementary file 1 [file DataSheet1.pdf]

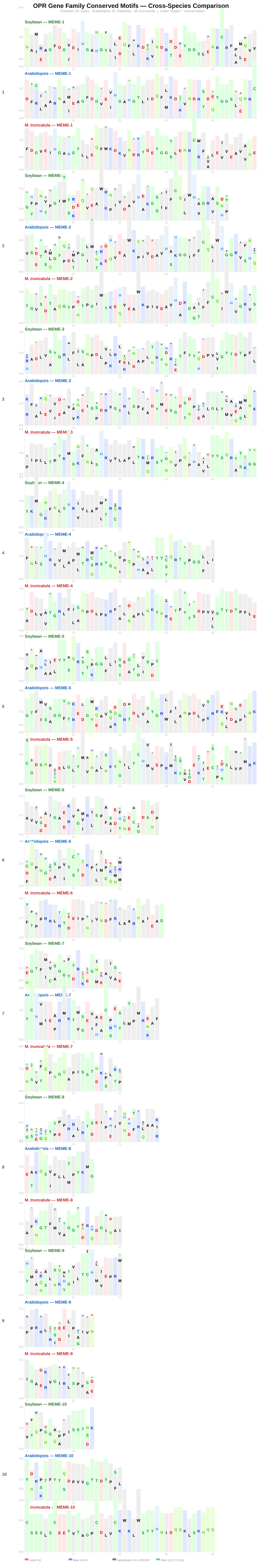
OPR Gene Family Conserved Motifs — Cross-Species Comparison
Soybean (G. max) · Arabidopsis (A. thaliana) · M. truncatula | Letter height = conservation
Soybean — MEME-1
Arabidopsis — MEME-1
M. truncatula — MEME-1
Soybean — MEME-2
Arabidopsis — MEME-2
M. truncatula — MEME-2
Soybean — MEME-3
Arabidopsis — MEME-3
M. truncatula — MEME-3
Soybean — MEME-4
Arabidopsis — MEME-4
M. truncatula — MEME-4
Soybean — MEME-5
Arabidopsis — MEME-5
M. truncatula — MEME-5
Soybean — MEME-6
Arabidopsis — MEME-6
M. truncatula — MEME-6
Soybean — MEME-7
Arabidopsis — MEME-7
M. truncatula — MEME-7
Soybean — MEME-8
Arabidopsis — MEME-8
M. truncatula — MEME-8
Soybean — MEME-9
Arabidopsis — MEME-9
M. truncatula — MEME-9
Soybean — MEME-10
Arabidopsis — MEME-10
M. truncatula — MEME-10
Acidic D,E
Basic K,R,H
Hydrophobic A,V,L,I,P,W,F,M
Polar G,S,T,Y,C,N,Q
